# KCTD12 promotes tumorigenesis by facilitating CDC25B/CDK1/Aurora A-dependent G2/M transition

**DOI:** 10.1038/onc.2017.287

**Published:** 2017-09-04

**Authors:** Y Zhong, J Yang, W W Xu, Y Wang, C-C Zheng, B Li, Q-Y He

**Affiliations:** 1 Key Laboratory of Functional Protein Research of Guangdong Higher Education Institutes, Institute of Life and Health Engineering, College of Life Science and Technology, Jinan University, Guangzhou, China; 2 Department of Pathology, Medical College, Jinan University, Guangzhou, China; 3 Institute of Biomedicine, Guangdong Provincial Key Laboratory of Bioengineering Medicine, National Engineering Research Center of Genetic Medicine, Jinan University, Guangzhou, China

## Abstract

Cell cycle dysregulation leads to uncontrolled cell proliferation and tumorigenesis. Understanding the molecular mechanisms underlying cell cycle progression can provide clues leading to the identification of key proteins involved in cancer development. In this study, we performed proteomics analysis to identify novel regulators of the cell cycle. We found that potassium channel tetramerization domain containing 12 (KCTD12) was significantly upregulated in M phase compared with S phase. We also found that KCTD12 overexpression not only facilitated the G2/M transition and induced cancer cell proliferation, but also promoted the growth of subcutaneous tumors and Ki-67 proliferation index in mice. Regarding the mechanism underlying these phenomena, cyclin-dependent kinase 1 (CDK1) was identified as an interacting partner of KCTD12 by immunoprecipitation and mass spectrometry analysis, which showed that KCTD12 activated CDK1 and Aurora kinase A (Aurora A) and that the effects of KCTD12 on CDK1 phosphorylation and cell proliferation were abrogated by cell division cycle 25B (CDC25B) silencing. In addition, Aurora A phosphorylated KCTD12 at serine 243, thereby initiating a positive feedback loop necessary for KCTD12 to exert its cancer-promoting effects. Furthermore, we analyzed the expression levels of various genes and the correlations between the expression of these genes and survival using tumor tissue microarray and Gene Expression Omnibus (GEO) data sets. The data showed that KCTD12 expression was significantly upregulated in cervical and lung cancers. More importantly, high KCTD12 expression was associated with larger tumor sizes, higher pathological stages and poor patient survival. Collectively, our study demonstrate that KCTD12 binds to CDC25B and activates CDK1 and Aurora A to facilitate the G2/M transition and promote tumorigenesis and that Aurora A phosphorylates KCTD12 at serine 243 to trigger a positive feedback loop, thereby potentiating the effects of KCTD12. Thus, the KCTD12-CDC25B-CDK1-Aurora A axis has important implications for cancer diagnoses and prognoses.

## Introduction

Cell cycle dysregulation is a common feature of human cancers that leads to at least two hallmarks of cancer development, namely, uncontrolled cell proliferation and genomic and chromosomal instability.^1, 2, 3^ It is well established that proper progression through the cell cycle is monitored by members of the cyclin-dependent kinase (CDK) family, whose activity is regulated by specific activators (cyclins) and inhibitors (Ink4 and Cip/Kip family members).^4^ Constitutive CDK activation causes a significant change in protein phosphorylation and drives tumor cell cycle progression, which results in unscheduled cell proliferation and tumorigenicity.^5, 6^ These findings suggest that CDKs may be a therapeutic target for the treatment of human cancer. Increasing amounts of evidence suggest that aberrant CDK activation is caused by various genetic and epigenetic events;^4, 7, 8^ however, the molecular mechanisms underlying these phenomena remain incompletely understood. Thus, studies identifying novel molecules and regulatory pathways that participate in regulating CDKs and the cell cycle are urgently needed.

Among the four sequential phases of cell cycle, the most important phases are S phase, in which DNA replication occurs, and M phase, in which the cell divides into two daughter cells. Comprehensive and systematic investigations of the mechanisms regulating cell cycle checkpoints in S phase and M phase may provide researchers with important clues enabling them to gain an extensive understanding of cell cycle regulation. Proteomics techniques have been widely used in cell cycle research,^9, 10, 11, 12, 13^ especially in studies attempting to identify phosphorylated proteins and protein complexes. In this study, we used stable isotope labeling with amino acids in cell culture (SILAC), a quantitative proteomics technique,^14^ in the cell synchronization model to systemically analyze the proteins that are differentially expressed between S phase and M phase and to identify novel regulators of cell cycle progression. Among the 54 proteins identified by the above analysis, potassium channel tetramerization domain containing 12 (KCTD12), a member of the KCTD family whose function in the cell cycle is unknown, drew our attention.

KCTD12 has two conserved binding domains, a BR-C, ttk and bab (BTB)/pox virus and zinc finger (POZ) domain rich in α-helices and β-folds at its N-terminal and a domain rich in β-folds at its C-terminal.^15^ *KCTD12* gene was firstly identified from the human fetal cochlear cDNA library,^16^ and its coding protein KCTD12 has been reported to induce GABA_B_ receptor-activated K^+^ current desensitization by binding to the βγ subunits of the activated G-protein, which interferes with activation of the effector K^+^ channel.^17, 18^ More importantly, KCTD12 overexpression and the correlation between KCTD12 overexpression and higher tumor grades have been reported in studies regarding gastrointestinal stromal tumors;^19^ however, KCTD12 has been shown to function as a tumor suppressor in studies regarding colon cancer.^20^ Thus, the role of KCTD12 in cancer remains unclear and controversial.

It has been documented that CDK1 phosphorylation is regulated by CDC25, Wee1 and Myt1. CDK1 activity is inhibited when the protein is phosphorylated by Wee1 and Myt1 during cell interphase but is activated and promotes cell cycle progression when it is dephosphorylated by CDC25 during G2/M transition.^21^ In addition, Aurora kinase A (Aurora A) can phosphorylate and activate CDC25 and then activate CDK1 to promote the rapid and timely entry of the cell into mitosis.^22^ CDK1 in turn initiates the entry of the cell into mitosis and the activation of Aurora A, thereby forming a positive-feedback loop in human cells.^23^ In the present study, immunoprecipitation coupled with proteomics analysis showed that KCTD12 interacts with CDK1 and cell division cycle 25B (CDC25B). Thus, the molecular mechanisms responsible for the regulation of KCTD12 by Aurora A, as well as the mechanisms by which KCTD12 exerts positive effects on Aurora A phosphorylation through CDK1 activation, were investigated. Furthermore, we performed *in vitro* and *in vivo* experiments to determine the function of KCTD12 in cell cycle regulation and tumorigenesis, as well as its clinical significance in cervical cancer and lung cancer.

## Results

### KCTD12 is a novel protein involved in cell cycle regulation

**Figure 1:**
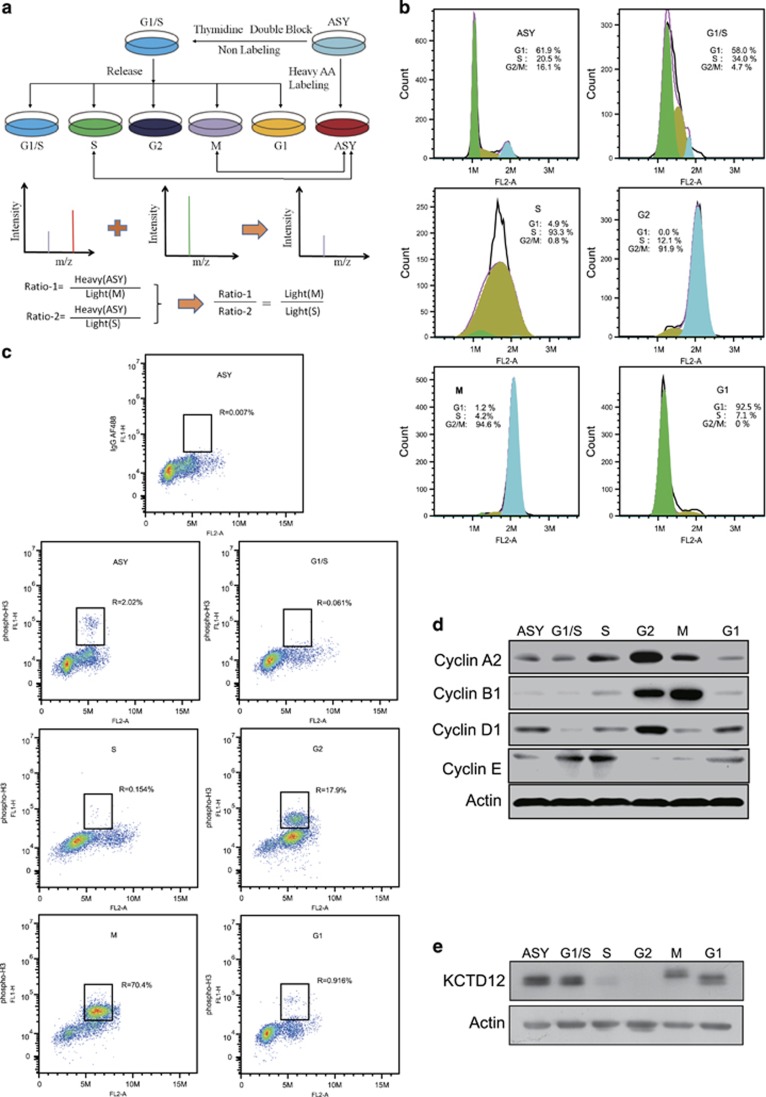
KCTD12 is differentially expressed between M phase and S phase in HeLa cells. (**a)** The workflow for the cell cycle synchronization and SILAC experiments. The cells were grown in ^13^C-Lysine medium to obtain heavy AA-labeled ASY cells or normal culture medium and synchronized to the G1/S, S, G2, M, and G1 phases using the double-block method with thymidine and nocodazole. Quantitative proteomics analysis was performed using a modified spike-in SILAC method to compare the protein profiles of the M and S phases. (**b)** Cell cycle synchronization efficiency was examined by flow cytometry. ASY: asynchronous cells. (**c**) Percentage of M phase cells were detected by flow cytometry with H3P antibody labeling. IgG AF488, negative control; ASY, asynchronous cells. (**d)** Validation of cell synchronization efficiency by western blotting analysis of the expression of specific cell cycle markers. ASY: asynchronous cells. (**e)** KCTD12 expression levels in different phases of the cell cycle were determined by western blotting.

To identify novel cell cycle regulators, we performed cell cycle synchronization using the double-block method with thymidine and nocodazole (Figure 1a). The flow cytometry results showed that the cell model was successfully established, with more than 90% of the HeLa cells synchronized at each stage (Figure 1b). In order to discriminate the M phase cells directly, flow cytometry assays were carried out with phospho-H3 (H3P) antibody, and the results showed that about 70% of the synchronized M phase cells were H3P-positive (Figure 1c). Considering the labeling efficiency of the antibody, we can conclude that >70% of the cancer cells were successfully synchronized to M phase. Cell synchronization efficiency was confirmed at the molecular level by western blotting analysis, whose results showed the expression levels of the markers of each cell cycle phase (Figure 1d). Using SILAC quantitative analysis, we determined that 54 proteins, including the mitosis-specific protein cyclin B1, were expressed at a level that was at least 1.5-fold higher in M phase than in S phase (Supplementary Table S1), suggesting that synchronized cells could be used as a cell model for identifying novel cell cycle regulators. Among these differentially expressed proteins, KCTD12, which has not been linked to cell cycle and has an unknown biological function, attracted our interest. To confirm our proteomics data, we performed western blotting, the results of which showed that KCTD12 expression levels varied cyclically during the cell cycle. More importantly, the results showed that KCTD12 expression decreased in S phase, was almost completely absent in G2 phase, and recovered in M phase (Figure 1e). We found that two bands of KCTD12 were detected, and the same phenomenon was also reported in previous published papers from other groups.^20, 24, 25^ Since cell cycle deregulation is a common feature of human cancer, we focused on the role of KCTD12 in cancer progression and the mechanisms underlying its effects in cancer in this study. To date, these mechanisms have not been elucidated.

### Clinical significance of KCTD12 in human cancer

**Figure 2:**
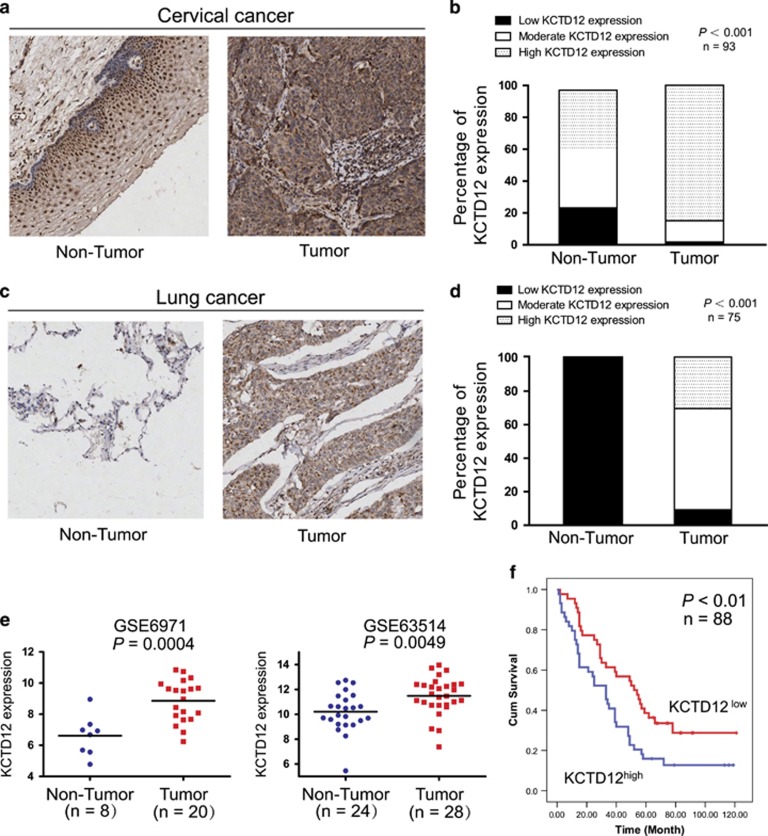
Clinical significance of KCTD12 in cervical and lung cancers. (**a)** Representative results of immunohistochemical staining for KCTD12 expression in cervical cancer tissues and adjacent non-tumor tissues. (**b)** Statistical analysis showed that KCTD12 expression was significantly increased in cervical cancer tissues compared with non-tumor tissues (*n*=93). (**c**) Representative results of immunohistochemical staining for KCTD12 expression in lung cancer tissues and paired non-tumor tissues. (**d)** KCTD12 expression levels were upregulated in lung cancer tissues compared with non-tumor tissues (*n*=75). (**e**) Analysis of *KCTD12* expression in cervical cancer and paired normal tissues using the GEO database. (**f)** Kaplan–Meier analysis of overall survival in 88 patients with lung cancer stratified according to their tumor KCTD12 expression levels.

To determine the clinical significance of KCTD12 in human cancer, we examined KCTD12 expression levels by immunohistochemistry using a tissue microarray containing 93 pairs of primary cervical cancer tissues and adjacent normal tissues. KCTD12 was localized in the cytoplasm of tumor cells, similar to its localization in colon cancer.^20^ We detected stronger KCTD12 cytoplasmic staining in 64 (68.8%) cervical cancer tissues than in corresponding normal tissues (Figure 2a). Notably, 84.9% (79/93) of tumor tissues showed high KCTD12 expression, whereas only 39.8% (37/93) of normal tissues showed high KCTD12 expression. In contrast, only 15.1% of tumor tissues displayed low-to-moderate KCTD12 expression, whereas 60.2% of normal tissues showed low-to-moderate KCDT12 expression (Figure 2b). The immunohistochemical analysis of KCTD12 expression in 75 pairs of lung cancer tissues and corresponding normal tissues showed the similar results (Figure 2c). The results of this analysis showed that majority of lung cancer tissues showed moderate-to-high KCTD12 expression, whereas all the normal tissues showed undetectable KCTD12 expression (Figure 2d). Analysis of the gene expression profiles of two cohorts of patients from the Gene Expression Omnibus (GEO) database also showed that KCTD12 expression is markedly upregulated in cervical cancer tissue compared with normal tissue (Figure 2e). Furthermore, to determine whether KCTD12 can be a prognostic marker for patients with cancer, we performed an immunohistochemical analysis of KCTD12 expression in a cohort of patients with lung cancer with survival data. We noted the existence of a significant correlation between tumor KCTD12 expression levels and tumor size, as well as pathologic T stage, in the 88 lung cancer patients who underwent pre-therapy surgery (Table 1). More importantly, we noted that patients with high tumor KCTD12 expression levels had significantly shorter survival (median survival=33.0 months) than patients with low tumor KCD12 expression levels (median survival=54.0 months). Moreover, log-rank analysis showed that high KCTD12 expression was significantly correlated with shorter survival (log-rank=6.897, *P*=0.0086; Figure 2f). Collectively, our results indicated that KCTD12 may play an important role in human cancer.

### KCTD12 promotes cancer cell proliferation

**Figure 3:**
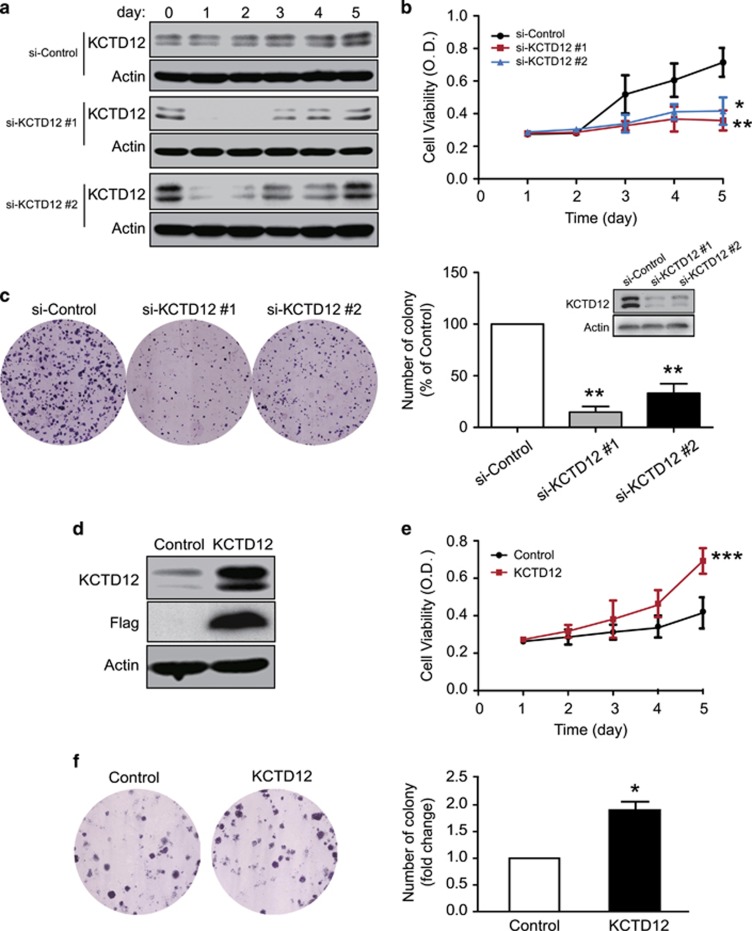
KCTD12 promotes cell proliferation in cervical cancer cells. (**a**–**c)** HeLa cells were transfected with a siRNA against KCTD12 (100 nM) or si-Control. Kinetic KCTD12 and actin expression levels were determined by western blotting (**a**), and cell proliferation and colony formation ability was examined by WST-1 (**b**) and colony formation assay (**c**). The western blotting in panel c was performed 24 h after the siRNA transfection. (**d**–**f)** HeLa cells transfected with a KCTD12-expressing plasmid (1 μg) or vector control were compared regarding KCTD12 expression (**d**), cell proliferation (**e**) and colony formation ability (**f**). Bars, s.d.; *P<0.05; ***P<0.001 compared with control cells.

We next studied the biological function of KCTD12 in cancer cells. WST-1 cell proliferation assay showed that KCTD12 knockdown significantly inhibited HeLa cell proliferation (Figures 3a and b). We performed colony formation assay to determine the effects of KCTD12 on cancer cell colony formation ability, the results of which showed that HeLa cells with KCTD12 knockdown formed fewer colonies than negative-control cells (Figure 3c). In addition, we found that KCTD12 overexpression had the opposite effect on HeLa cell proliferation (Figures 3d and e) and colony formation (Figure 3f). Moreover, we confirmed the cancer-promoting effects of KCTD12 in lung cancer A549 cells by performing gain- and loss-of-function experiments (Supplementary Figures S1a–d).

### KCTD12 interacts with CDC25B and activates CDK1 signaling

**Figure 4:**
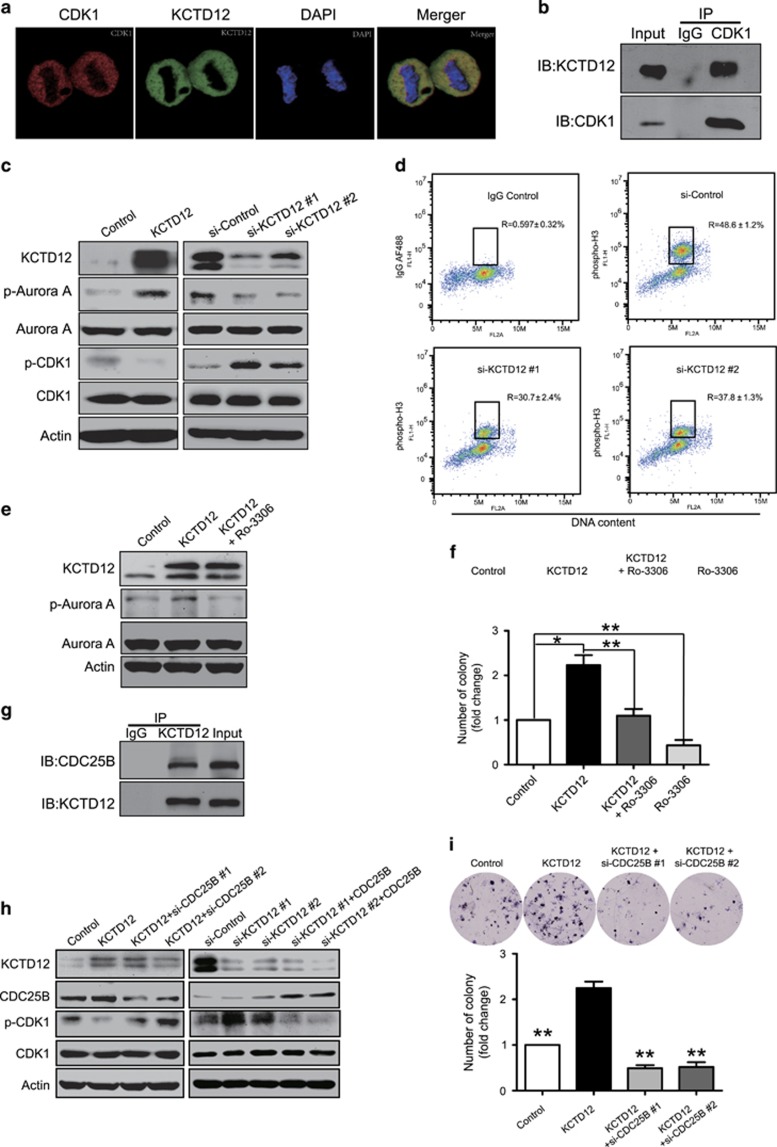
KCTD12 interacts with CDC25B to promote G2/M transition and cell proliferation by dephosphorylating CDK1. (**a**) Confocal immunofluorescence analysis of KCTD12 and CDK1 localization in HeLa cells. KCTD12, green; CDK1, red; DAPI was used to label the nuclei; the cells presented are at metaphase. (**b**) HeLa cells were synchronized to M phase, and whole-cell lysates were immunoprecipitated with a CDK1 antibody, and then KCTD12 expression was detected by western blotting. (**c**) HeLa cells were transfected with KCTD12-expressing plasmid (1 μg, 24 h, left panel) or the si-KCTD12 (100 nM, 24 h, right panel), and p-Aurora A, Aurora A, p-CDK1 and CDK1 expression levels in the experimental group were compared with those in the control group by western blotting. Actin was included as a loading control. (**d**) Knockdown of KCTD12 decreased the percentage of M phase cells. The cells labeled with IgG AF488 were used as negative control. (**e)** Inhibiting CDK1 activity with Ro-3306 (30 nM, 24 h) attenuated the effect of KCTD12 (1 μg, 24 h) on Aurora A phosphorylation. (**f)** Ro-3306 decreased KCTD12-induced cell proliferation. HeLa cells were treated with Ro-3306 (30 nM, 24 h) or transfected with KCTD12-expressing plasmid (1 μg, 24 h) alone, or the combination, and colony-formation ability was compared. (**g**) The interaction between KCTD12 and CDC25B was examined by Co-IP assay in the HeLa cells synchronized at M phase. (**h)** KCTD12-expressing plasmid (1 μg) or vector control were co-transfected into HeLa cells with the siRNA against CDC25B (100 nM) or the si-Control (left panel); on the contrary, the siRNA against KCTD12 (100 nM) or si-Control was transfected into HeLa cells with or without CDC25B-expressing plasmid (right panel). Cells were harvested 24 h after transfection, and expression levels of KCTD12, CDC25B, p-CDK1 and CDK1 were determined by western blotting. (**i**) HeLa cells were co-transfected with KCTD12-expressing plasmid or vector control and si-CDC25B or si-Control, and the ability of the cells to form colonies was compared by colony formation assay. Bars, s.d.; *P<0.05; **P<0.01; ***P<0.001 compared with cells transfected with the KCTD12-expressing plasmid.

To explore the mechanisms of action underlying the effects of KCTD12 in cancer, we performed immunoprecipitation coupled with liquid chromatography tandem mass spectrometry (LC-MS) to identify its interacting proteins (Supplementary Table S2). Among the proteins identified, CDK1, one of the most important regulators of cell cycle progression, drew our attention. We performed immunofluorescence staining and Co-IP to confirm the results of the proteomics analysis. The results of this analysis showed that KCTD12 and CDK1 co-localized in the cytoplasm of metaphase cells (Figure 4a) and that KCTD12 could interact directly with CDK1 in M phase cells (Figure 4b). We also found that KCTD12 overexpression activated CDK1, a finding supported by our observation of CDK1 dephosphorylation at Thr14/Tyr15 (p-CDK1; Figure 4c, left). In addition, KCTD12 knockdown significantly increased p-CDK1 expression levels (Figure 4c, right). In order to examine whether KCTD12 is important for G2/M transition, we carried out the flow cytometry assays in KCTD12-knockdown HeLa cells. The data showed that KCTD12 silencing significantly decreased the percentage of M phase cells (Figure 4d). Interestingly, Aurora A, one of the downstream targets of CDK1,^26^ was phosphorylated at Thr288 in KCTD12-overexpressing cells; whereas KCTD12 knockdown exerted the opposite effect (Figure 4c). As shown in Figure 4e, the effects of KCTD12 on Aurora A phosphorylation could be abrogated by CDK1 blockade with an ATP-competitive and selective CDK1 inhibitor Ro-3306, suggesting that KCTD12 may phosphorylate Aurora A through CDK1. Moreover, the results of the colony formation assay indicated that CDK1 activity was required for the cancer-promoting effects of KCTD12 (Figure 4f). Furthermore, we investigated the molecular mechanisms underlying the effects of KCTD12 on CDK1 activity. Given that CDK1 is dephosphorylated at Thr14/Tyr15 by CDC25B, an important cell cycle protein, when cells enter into mitosis,^27, 28^ we postulated that CDC25B may participate in KCTD12-mediated CDK1 regulation. Our hypothesis was strongly supported by the data showing that KCTD12 directly interacted with CDC25B (Figure 4g). In addition, the CDK1 dephosphorylation induced by KCTD12 was significantly attenuated by CDC25B knockdown (Figure 4h, left panel), and conversely, the phosphorylation of CDK1 induced by KCTD12 silencing could also be eliminated by CDC25B overexpression (Figure 4h, right panel). Functionally, CDC25B silencing significantly abrogated KCTD12-induced cell proliferation in HeLa cells (Figure 4i). Taken together, our results demonstrate that KCTD12 may form a complex with CDC25B and CDK1 and thus activate CDK1 and promote G2/M transition and cell proliferation.

### KCTD12 is phosphorylated by Aurora A at serine 200 and serine 243

**Figure 5:**
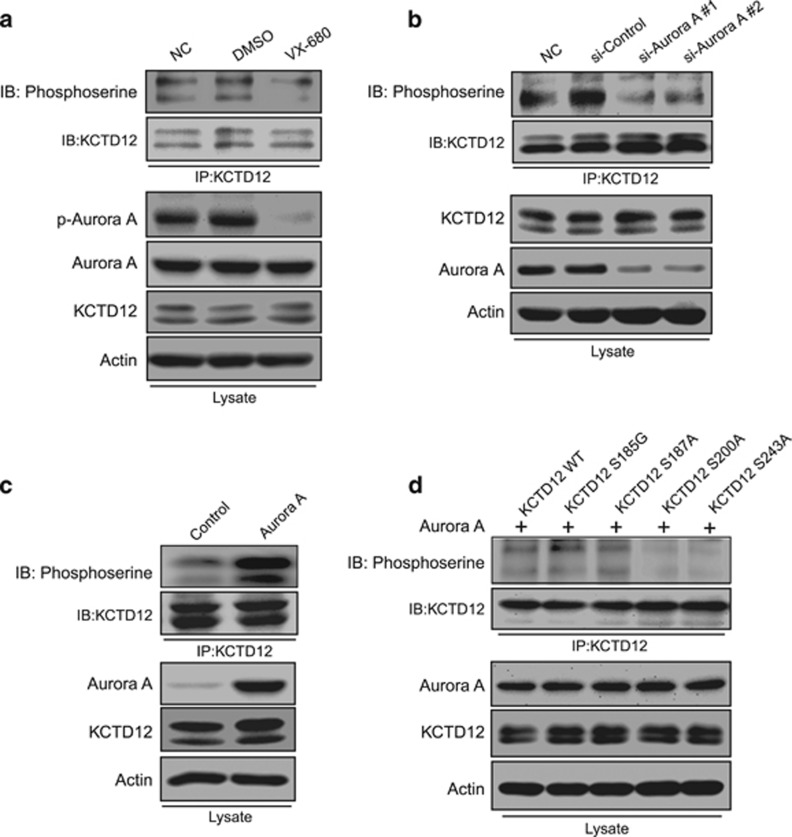
KCTD12 is phosphorylated by Aurora A at serine 200 and serine 243. (**a**) HeLa cells were treated with DMSO or VX-680 (0.5 μM, 24 h). The cell lysates were immunoprecipitated with a KCTD12 antibody, and the bound proteins were analyzed by immunoblotting with a phosphoserine antibody. p-Aurora A and actin expression levels were determined in the whole-cell lysates. (**b**) Comparisons of KCTD12 phosphorylation levels in HeLa cells transfected with the siRNA against Aurora A (100 nM, 24 h) or the control siRNA with those in untransfected HeLa cells were assessed by immunoprecipitation and western blotting. (**c**) Aurora A (1 μg, 36 h) overexpression in HeLa cells had a positive effect on KCTD12 phosphorylation. (**d**) HeLa cells were co-transfected with a plasmid expressing Aurora A and a plasmid expressing wild-type KCTD12 or an S185G, S187A, S200A, S243A mutant. KCTD12 phosphorylation levels were detected by immunoprecipitation and western blotting. Note that KCTD12 could not be phosphorylated by Aurora A when the serine 200 or serine 243 site was mutated.

We next studied the mechanisms responsible for an upward shift of the KCTD12 band in M phase (Figure 1d). The data showing that the band of KCTD12 was significantly moved down when the cell lysates were treated with calf intestinal alkaline phosphatase (CIP; Supplementary Figure S2a) led us to hypothesize that the phenomenon may be attributable to KCTD12 phosphorylation.^29^ We retrieved a database containing data regarding protein phosphorylation during cell cycle progression^30^ and found that KCTD12 can be phosphorylated by Aurora A. To verify that KCTD12 phosphorylation in M phase was regulated by Aurora A, we performed an experiment involving a selective Aurora kinase inhibitor (VX-680). The results of the experiment showed that KCTD12 phosphorylation, which was indicated by the band location, was abolished by treatment with VX-680 (Supplementary Figure S2b). KCTD12 phosphorylation and the mechanisms underlying the process were verified by immunoprecipitation using a KCTD12 antibody and a subsequent western blotting analysis using phosphoserine antibody. As expected, both VX-680 treatment (Figure 5a) and Aurora A knockdown (Figure 5b) significantly decreased KCTD12 phosphorylation levels in HeLa cells. Since KCTD12 was predicted to be phosphorylated by Aurora A at 4 serine sites, we constructed 4 plasmids with S185G, S187A, S200A and S243A mutations to determine which serine site was phosphorylated by Aurora A. We co-transfected HeLa cells with Aurora A and KCTD12 wild-type or mutant plasmids. The results of the experiment showed that Aurora A significantly increased KCTD12 phosphorylation levels (Figure 5c) and that KCTD12 could still be phosphorylated when S185 or S187 was mutated but could not be phosphorylated when S200 or S243 was mutated (Figure 5d). Therefore, we concluded that Aurora A can phosphorylate KCTD12 at S200 and S243 but not at the other 2 serine sites. Taken together, our findings indicate that KCTD12 is regulated by Aurora A through phosphorylation.

### KCTD12 phosphorylation at serine 243 is necessary for the promotion of cancer cell proliferation and tumorigenesis

**Figure 6:**
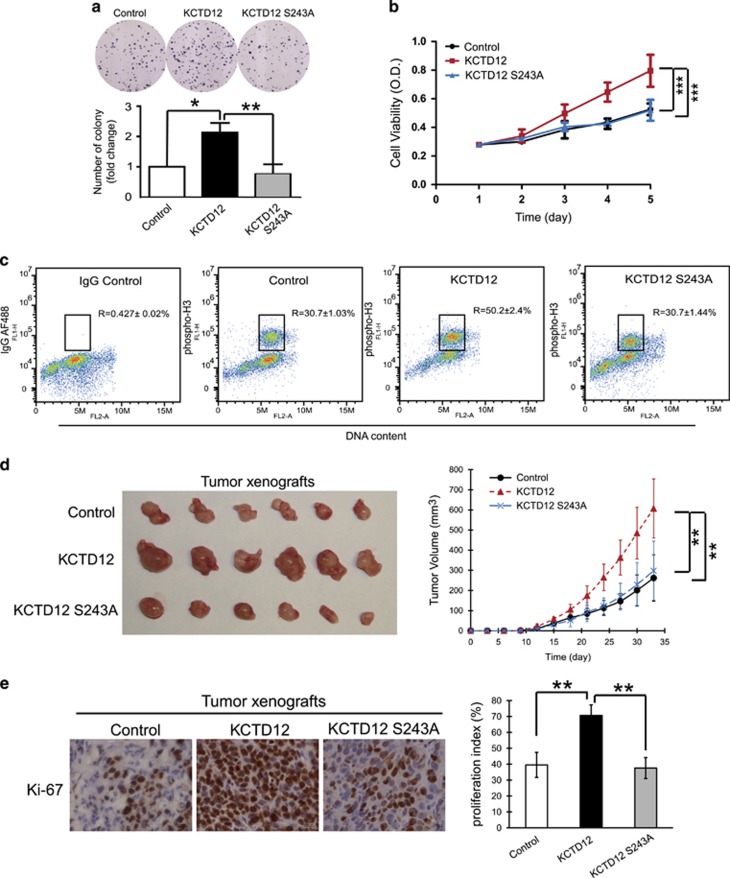
KCTD12 phosphorylation at Ser243 is necessary for cervical cancer cell proliferation and tumorigenesis. (**a**–**c)** HeLa cells were transfected with a plasmid expressing wild-type KCTD12, an S243 mutant or an empty vector (1 μg, 24 h). The ability of the cells to proliferate and form colonies was compared among the three groups by colony formation (**a**) and WST-1 assays (**b**), and flow cytometry was performed to determine the percentage of cells at M phase in the three groups of cells transfected with the indicated plasmids (**c**). (**d**) Images of the tumors (left panel) and the growth curves (right panel) of the subcutaneous tumors formed by HeLa-KCTD12, HeLa-KCTD12 S243A, or vector control cells in nude mice (*n*=6). (**e**) Immunostaining for Ki-67 in tumor xenografts from the 3 groups (left panel) and quantification of the tumor proliferation index (right panel). Bars, s.d.; *P<0.05; **P<0.01; ***P<0.001 compared with controls unless otherwise indicated.

To determine whether KCTD12 phosphorylation at serine 200 and serine 243 by Aurora A is required for the cancer-promoting effects of KCTD12, we performed a series of *in vitro* and *in vivo* experiments. First, we transfected HeLa cells with a plasmid expressing wild-type KCTD12, a KCTD12 S200A mutant, a KCTD12 S243A mutant, or a vector control, and analyzed the colony formation ability of these cells. The results showed that overexpression of wild-type KCTD12 or the KCTD12 S200A mutant enhanced colony formation ability in HeLa cells, while overexpression of the KCTD12 S243A mutant failed to exert this effect (Figure 6a and Supplementary Figure S3), suggesting that phosphorylation of KCTD12 at serine 200 is not required for its effects on cancer cell proliferation. As shown in Figure 6b, the WST-1 data showing that wild-type KCTD12 but not KCTD12 S243A promoted HeLa cell proliferation confirmed that serine 243 plays an important role in the biological function of KCTD12. Regarding the mechanism underlying this phenomenon, we found that overexpression of wild-type KCTD12, but not the S243A mutant, could significantly increase the percentage of cells entering M phase (Figure 6c).These data indicated that KCTD12 facilitated the G2-to-M phase transition and that serine 243 is necessary for this process. Furthermore, to examine the role of KCTD12 in facilitating tumorigenicity *in vivo*, we subcutaneously injected HeLa cells ectopically expressing wild-type KCTD12, an S243A mutant, or a vector control into nude mice. The results of this experiment showed that HeLa cells stably expressing wild-type KCTD12 formed larger tumors than cells expressing the vector control (607.1±146.8 vs 261.9±115.0 mm^3^; *P*<0.01); however, the stimulatory effects of KCTD12 on tumor growth were abrogated when serine 243 in KCTD12 was mutated (Figure 6d). Moreover, comparisons of the tumor xenografts by immunohistochemistry showed that the stimulatory effects of KCTD12 on tumor growth were attributable to increases in cell proliferation, a finding supported by our observation of a higher Ki-67 proliferation index (the mean index increased from 39.5±7.9% in the vector control group to 70.7±6.6% in the wild-type KCTD12 group; *P*<0.01; Figure 6e). Collectively, these data showed that KCTD12 drove cell cycle progression and tumorigenicity and that serine 243 is crucial for these effects.

### KCTD12 facilitates the interaction between CDC25B and CDK1

**Figure 7:**
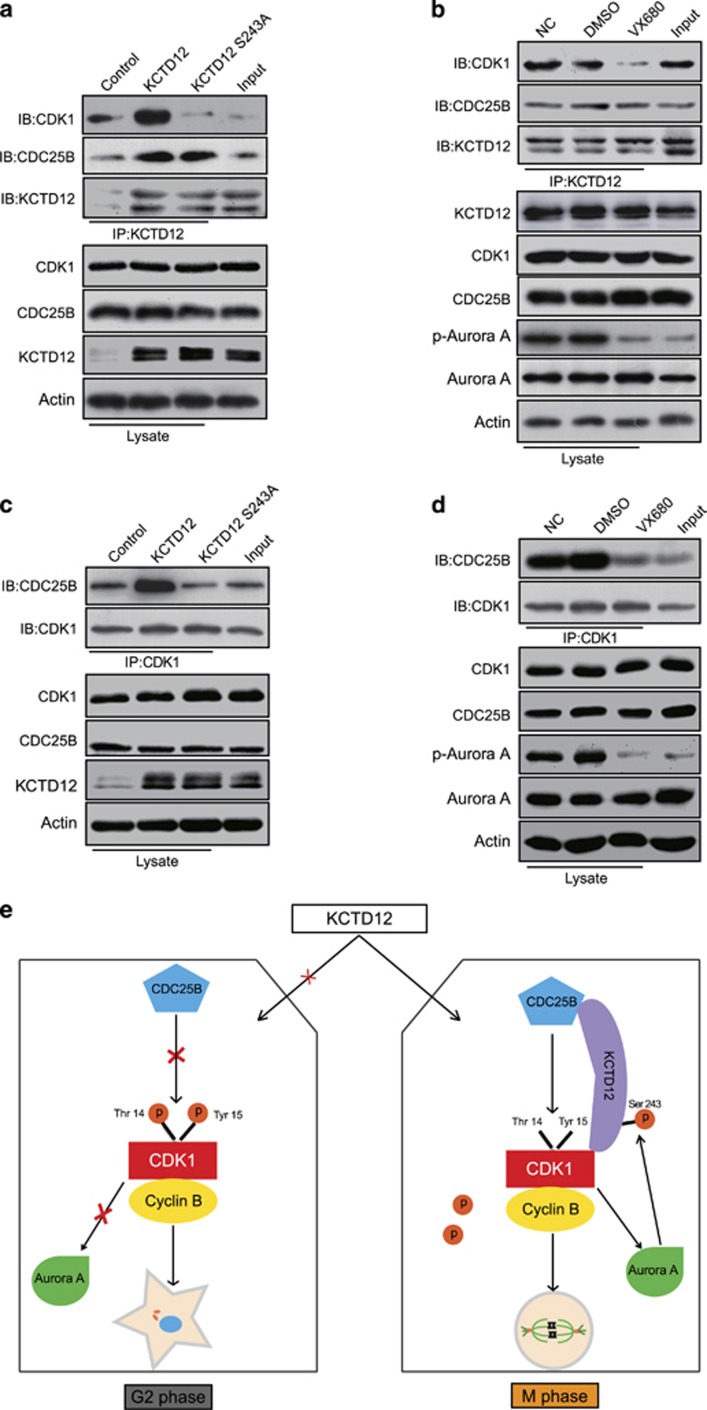
KCTD12 interacts with CDC25B and CDK1 to form a positive feedback loop and facilitate the G2/M transition. (**a**, **b**) KCTD12 S243 phosphorylation was necessary for its interaction with CDK1 but not CDC25B. The HeLa cells transfected with KCTD12 wild-type plasmid or S243A mutant plasmid (1 μg, 24 h) were used to perform the immunoprecipitation assays (**a**), and the cells treated with VX-680 (0.5 μM, 24 h) were used to determine the impact of Aurora A on the interaction of KCTD12 and CDC25B or CDK1 (**b**). (**c**) The HeLa cells were transfected with wild type KCTD12- or S243A mutant-expressing plasmid (1 μg, 24 h), and CDC25B expression was analyzed in the CDK1 immunoprecipitates by immunoblotting. Expression levels of CDK1, CDC25B, KCTD12 and Actin were also determined in the whole cell lysates. The cells used for immunoprecipitation were synchronized to M phase. (**d**) The interaction of CDC25B and CDK1 in the HeLa cells treated with DMSO or VX-680 (0.5 μM, 24 h) were assessed by immunoprecipitation and western blotting. Cells were synchronized to M phase for immunoprecipitation. (**e**) Schematic diagram summarizing how the KCTD12-CDC25B-CDK1-Aurora A positive-feedback loop facilitates cell entry into mitosis.

We next investigated the mechanisms how phosphorylation of KCTD12 at serine 243 is important for its role in promoting cell proliferation. The immunoprecipitation experiments showed that the ability of KCTD12 to interact with CDK1 was significantly abolished when serine 243 of KCTD12 was mutated (Figure 7a), and the same phenomenon was observed when the cells were treated with the Aurora A inhibitor VX-680 (Figure 7b), while the binding of KCTD12 to CDC25B was not disrupted under either condition. As the interaction between CDC25B and CDK1 is necessary for G2/M transition, and the above findings indicated that the phosphorylation of KCTD12 at serine 243 is important for its effects on G2/M transition and cell proliferation (Figure 6), we further examined the impacts of KCTD12 and its S243 site on the interaction of CDC25B and CDK1. HeLa cells were transfected with the plasmid expressing wild type KCTD12 or S243A mutant, and the expression level of CDC25B was determined in the CDK1 immunoprecipates. The results demonstrated that wild type KCTD12, but not S243A mutant, facilitated the interaction of CDC25B and CDK1 (Figure 7c). In addition, we explored the effects of Aurora A on the interaction of CDC25B and CDK1. As indicated in Figure 7d, the interaction between CDK1 and CDC25B was significantly decreased in the cells treated with VX-680. Taken together, our study indicates that KCTD12 exerts its effects in cancer by forming a complex with CDC25B and CDK1, and the phosphorylation of KCTD12 at serine 243 is necessary for the interaction of KCTD12 and CDK1, but it is not a crucial factor for the interaction with CDC25B. The phosphorylation of KCTD12 at S243 could also facilitate the interaction of CDC25B and CDK1 to activate CDK1 and its downstream target, Aurora A, which in turn phosphorylates KCTD12 at serine 243 to activate KCTD12, thereby initiating a positive feedback loop that facilitates cell entry into mitosis and promotes cancer cell proliferation (Figure 7e).

## Discussion

As one of the hallmarks of cancer, cell cycle dysregulation leads to tumorigenesis and cancer progression. However, the mechanisms underlying this phenomenon remain to be elucidated. Studies systemically identifying and characterizing novel regulators of cell cycle progression are urgently needed. In this study, successful establishment of the cell synchronization model enabled us to purify the cancer cells at each phase and compare their protein profiles using SILAC and LC-MS/MS. By identifying cyclin B1, the mitosis-specific protein,^31^ as one of the proteins expressed at a higher level in M phase than in S phase, we demonstrated that this screening strategy is an effective tool for identifying proteins that are differentially expressed in each cell cycle phase, as well as novel regulators of cell cycle procession. Here we provide the first evidence indicating that KCTD12 can facilitate cancer cell entry into M phase and promote cell proliferation and tumorigenesis.

CDK1 activation promotes mitotic spindle formation and centrosome separation and ultimately induces cell mitosis.^32, 33^ It is well known that CDK1 can be activated by CDC25B through dephosphorylation at its Thr14/Tyr15 site,^34^ but it is unclear whether other molecules are involved in this process. In present study, by performing immunoprecipitation and mass spectrometry, we identified CDK1 as an interacting partner of KCTD12, which can dephosphorylate and activate CDK1 and thus promote cell proliferation. Moreover, we found that KCTD12 bound to CDC25B and that CDC25B knockdown significantly abrogated the effects of KCTD12 on CDK1 and the malignant phenotype in cancer cells (Figure 4), suggesting that KCTD12 may interact with both CDK1 and CDC25B to form a complex and thus play an important role in cell cycle regulation and cancer development. Our results indicate that aberrant CDK1 signaling activation plays a role in cancer cell proliferation and may thus be a potential therapeutic target in the treatment of human cancer. Recent advances in chemistry biology and detection technology have been found to be instrumental in identifying inhibitors of protein binding.^35^ As we have uncovered evidence that the interaction between KCTD12 and CDK1 play an important role in cancer progression, our next step is to screen these protein binding inhibitors to determine their usefulness in cancer therapy.

Aurora A modifies the structure and function of the cytoskeleton and chromosomes and thus regulates cell cycle progression.^36^ Aurora A also interacts with BRCA1 to induce breast tumorigenesis^37^ and regulates the transcription of human telomerase reverse transcriptase through c-myc pathways in ovarian and breast epithelial carcinomas.^38^ Phosphorylation is the most common post-translational modification of proteins.^39^ The results of our immunoprecipitation and mutation experiments indicated that KCTD12 can be phosphorylated by Aurora A at serine 200 and serine 243, and the results of our functional assays showed that serine 243 but not serine 200 is required for the cancer-promoting effects of KCTD12 (Figure 6, Supplementary Figure S3). Interestingly, Aurora A, a downstream target of CDK1, was found to be phosphorylated by KCTD12 (Figure 4), a finding indicative of the existence of a positive regulatory loop in which KCTD12 facilitates the entry of cancer cells into M phase and promotes cancer cell proliferation by activating CDK1 signaling, and Aurora A phosphorylates KCTD12 at serine 243. This feedback loop is necessary for KCTD12 to regulate cell cycle progression and tumorigenesis.

Studies aiming to develop effective diagnostic and prognostic biomarkers for cancer are urgently needed. In this study, we found that KCTD12 plays an important role in cell cycle regulation and tumorigenesis in cervical and lung cancers and report for the first time that KCTD12 is frequently upregulated in cervical and lung cancers. Our findings strongly suggest that KCTD12 may be a useful diagnostic biomarker for these cancers. More importantly, higher KCTD12 expression in primary tumors is significantly correlated with unfavorable tumor stages and shorter survival in patients with lung cancer. To determine the clinical significance of KCTD12 in cervical cancer, we searched for a tissue microarray with survival data. Unfortunately, cervical cancer cohorts with survival data are lacking. Cervical cancer is the third most common cancer among women worldwide and leads to 275 000 deaths every year.^40^ Our study demonstrated the prognostic value of KCTD12 in lung cancer; however, the potential of KCTD12 as a prognostic marker in cervical cancer warrants further investigation.

## Materials and methods

### Cell lines and drugs

Human cervical cancer HeLa cells and lung cancer A549 cells (ATCC, Rockville, MD, USA) were maintained in Dulbecco's modified Eagle's medium supplemented with 10% fetal bovine serum (Thermo Fisher Scientific, Waltham, MA, USA) at 37 °C with 5% CO_2_. The cell lines were authenticated by short tandem repeat profiling, and tested for mycoplasma contamination. VX-680 and Ro-3306 were purchased from Selleck Chemicals (Huston, TX, USA) and dissolved in dimethyl sulfoxide (DMSO).

### Cell cycle synchronization

We employed the double-block method with thymidine and nocodazole (Sigma, San Francisco, CA, USA) to develop the cell cycle synchronization model.^41^ For the first block, HeLa cells in exponential phase were treated with 2.5 mM thymidine for 18 h and then washed with PBS and released in medium for 10 h. For the second block, the cells were treated with 2.5 mM thymidine for 16 h. Some G1/S phase cells were subsequently collected. The remaining cells were released again, and S phase and G1 phase cells were collected 4 and 15 h later, respectively. G2 phase cells were collected upon being released and treated with nocodazole (100 ng/ml) for 8 h, and M phase cells were collected upon being released and treated with nocodazole for 10 h.

### SILAC labeling and LC-MS/MS analysis

Normal Dulbecco's modified Eagle's medium deficient in arginine (R) and lysine (K) was supplemented with L-[13C6, 15N4] arginine (R10) and L-[13C6] lysine (K6) for ‘heavy’ labeling, whereas L-[12C6,14N4] arginine (R0) and L-[12C6,14N2] lysine (K0; Thermo Fisher Scientific) were used for ‘light’ labeling, as described previously.^42, 43^ Equal amounts of ‘light’ and ‘heavy’ proteins were subsequently mixed and loaded onto SDS–PAGE gels for pre-separation. After in-gel digestion, the peptide mixtures were analyzed by reverse-phase LC-MS/MS.

### Plasmids, siRNAs and transfection

Flag-KCTD12 and Aurora A plasmids were generated by PCR amplification of sequences obtained from a colon cancer cDNA library and cloned into a pCMV-N-flag and pcDNA3.1 vector, respectively. The following primers were used to generate the KCTD12 and Aurora A plasmids: KCTD12: forward (5′-CCGGAATTCCACCTCTCTGTCATGGCTCT-3′) and reverse (5′-CTGCAGAGAACTCAGCACCAAG-3′); Aurora A: forward (5′-CGCGGATCCATGGACCGATCTAAAGAAAACTGCA-3′) and reverse (5′-CGCGATATCCTACTTGTCATCGTCGTCCTTGTAGTCAGACTGTTTGCTAGCTGATTCT-3′). Transfection was performed using Lipofectamine^TM^ 3000 reagent (Thermo Fisher Scientific, Waltham, MA, USA), according to the manufacturer’s recommendations. HeLa cells stably expressing wild-type KCTD12, KCTD12 S243A mutants or empty vectors were established with approximately 2 weeks of G418 selection. The siRNA sequences used in this study are listed in Supplementary Table S3.

### Site-directed mutagenesis

A site-directed mutagenesis kit (Agilent Technologies, Santa Clara, CA, USA) was used to generate KCTD12 S243A, S185G, S187A and S200A mutant constructs for the creation of a pCMV-N-flag-KCTD12 vector, according to the manufacturer’s instructions. The primers used for these experiments are listed in Supplementary Table S4.

### Western blotting

The cell pellets were suspended in lysis buffer (Cell Signaling Technology, Danvers, MA, USA) and incubated on ice for 30 min. The cell lysates were subsequently centrifuged at 14000 g for 30 min at 4 °C, after which supernatant was mixed with loading buffer and boiled for 5 min at 95 °C before being loaded onto a sodium dodecyl sulfate (SDS) polyacrylamide gel for electrophoresis. The proteins were subsequently transferred to polyvinylidene fluoride (PVDF) membranes (Millipore, Billerica, MA, USA). After being blocked with 5% fat-free milk in Tris-buffered saline-Tween 20 (TBST), the membranes were probed with the appropriate primary antibodies, followed by the corresponding horseradish peroxidase (HRP)-conjugated secondary antibodies (Cell Signaling Technology). The signals were detected by Clarity Western ECL Substrate (Bio-Rad, Hercules, CA, USA) and visualized by exposure to autoradiographic film. Antibodies against the following proteins were used for the experiment: KCTD12 (#I5523-1-AP) and cyclin A2 (# 66391-1-Ig), which was obtained from Proteintech Group (Chicago, IL, USA); andcyclin B1 (#sc-7393), cyclin D1 (#sc-8396), cyclin E (#sc-377100), CDK1 (#sc-954), p-CDK1 (#sc-12340-R), and actin (#sc-8432), which were obtained from Santa Cruz Biotechnology (Santa Cruz, CA, USA); and CDC25B (#ab124819), Aurora A (#ab1287) obtained from Abcam (Cambridge, MA, USA).

### Flow cytometric cell cycle analysis

The cells were fixed with 70% alcohol for 1 h at −20 °C and then stained with propidium iodide (PI) staining buffer (PBS containing 33 μg/ml PI, 0.13 mg/ml RNaseA, 10 mM EDTA, 0.5% TritonX-100) for 10 min at room temperature. Cell cycle analysis was performed on a BD Accuri C6 Analyzer (BD Biosciences, San Jose, CA, USA). Flow cytometry analyses of the cells stained with phospho-H3 AF488 antibody (#3465; H3P, Cell Signaling Technology) were performed as described previously.^44^ Briefly, the fixed cells were treated with 0.25% Triton X-100 on ice for 15 min, and then incubated with H3P antibody (1:1600 in 1% BSA) for 3 h at room temperature. The H3P antibody-labeled cells were subsequently stained with PI and analyzed on a flow cytometer.

### WST-1 assay

Cell viability was measured using a WST-1 Cell Proliferation and Cytotoxicity Assay Kit (Beyotime Biotechnology, Shanghai, China). Briefly, the cells were seeded in 96-well plates at an approximate density of 2000 cells were seeded per well. At the indicated time points, 10 μl of WST-1 reagent was added to the wells. The cells were subsequently incubated for 4 h at 37 °C. The absorbance was measured at a wavelength of 450 nm.

### Colony-formation assay

Colony-formation assay was performed as described previously.^45^ Briefly, the cells were seeded in six-well plates at an approximate density of 500 cells per well. After 14 days, the cells were fixed with methanol and stained with 0.1% crystal violet. The number of colonies was counted for analysis.

### Immunofluorescence

The cells were fixed in 4% paraformaldehyde and permeabilized with 0.1% TrintonX-100 for 10 min before being blocked with 10% goat serum for 2 h. The cells were then incubated with primary antibodies against the indicated proteins (KCTD12 and CDK1) overnight at 4 °C before being washed 3 times × 5 min with 1% TBST. The cells were subsequently incubated with the appropriate fluorescent secondary antibodies for 2 h at room temperature before being counterstained with 40,6-diamidino-2-phenylindole (DAPI, Thermo Fisher Scientific, Waltham, MA, USA) and observed by laser scanning confocal microscopy (Carl Zeiss AG, Jena, Germany).

### Co-immunoprecipitation (Co-IP)

The cell lysates were pre-washed with IgG (Santa Cruz Biotechnology) and protein A/G Sepharose beads (Invitrogen, Gaithersburg, MD, USA) for 1 h at 4 °C, and the cell supernatants were incubated with the appropriate primary antibody overnight at 4 °C before being incubated with protein A/G Sepharose beads for 4 h. The beads were washed thrice with lysis buffer and eluted in 2 × SDS/PAGE loading buffer for immunoblotting. For LC-MS/MS analysis, the lanes on the silver-stained gels were cut into several bands and in-gel digested, after which the peptide mixtures were analyzed by LC-MS/MS.

### Tumorigenicity in nude mice

The *in vivo* tumorigenesis experiment was performed as described previously.^46, 47^ Female BALB/c nude mice aged 6–8 weeks were maintained under standard conditions and cared for according to the institutional guidelines for animal care. Briefly, HeLa cells stably overexpressing wild-type KCTD12, mutant KCTD12, or vector controls were suspended in a 1:1 mixture of PBS/Matrigel and then subcutaneously injected into the flanks of the mice. Tumor size was measured with calipers every three days, and tumor volume was calculated using the following equation: *V*=(length × width^2^)/2. All animals were sex- and age-matched in the animal experiments, and littermates were used. The investigators were not blinded to the experimental groups. All animals were killed at the end of the study, and their tumors were collected for further analysis. All the animal experiments were approved by the Ethics Committee for Animal Experiments of Jinan University.

### Immunohistochemistry and staining evaluation

Immunohistochemistry was performed as described previously.^48^ A human cervical cancer tissue microarray containing 93 pairs of cervical cancer tissues and adjacent normal tissues (Shanghai Outdo Biotech, Shanghai, China), a human lung cancer tissue microarray containing 75 pairs of lung cancer tissues and adjacent normal tissues (Shanghai Outdo Biotech), and a human lung cancer tissue microarray containing 88 lung cancer tissue samples with survival data (Shanghai Outdo Biotech), as well as tumor xenograft sections, were deparaffinized in xylene, rehydrated in a graded series of ethanol solutions and processed for immunohistochemistry. After the slides were subjected to antigen retrieval and blocked with normal serum, they were incubated with a KCTD12 or Ki-67 antibody (Dako, Mississauga, ON, Canada) overnight at 4 °C, washed with PBS, and then incubated with the appropriate peroxidase-conjugated secondary antibody (Dako). Immunostaining was visualized using 3,3’-diaminobenzidine (Dako), which served as a chromogen, and the sections were counterstained with hematoxylin. The investigators were blinded to sample identity in the assessment of Ki-67 in tumor xenograft sections. KCTD12 immunostaining was evaluated based on scores representing the percentage of positively stained tumor cells and the staining intensity grade. These scores were determined by two independent pathologists. The percentages of positively stained tumor cells were scored according to the following scale: 0, <10% 1, 10–30% 2, 30–50% 3, 50–80% and 4, 80–100% and the staining intensities were classified into the following four categories: 0, no staining; 1, weak staining; 2, moderate staining; 3, strong staining. Ki-67 staining was assessed as described previously.^49^

### Statistical analysis

The results were analyzed using GraphPad PRISM software (GraphPad Software Inc., San Diego, CA, USA), and the data from different experiment were compared by Student’s *t*-test and expressed as the mean±s.d. Sample size in animal experiments was chosen on the basis of literature documentation of similar well-characterized experiments, and no statistical method was used to predetermine sample size. Pearson’s chi-square test was performed to analyze the association between KCTD12 expression levels and categorical clinicopathological variables. Survival analysis was performed using the Kaplan-Meier method with the log-rank test. *P*<0.05 was considered statistically significant.

## Figures and Tables

**Table 1:** Correlation between KCTD12 expression levels and clinicopathological parameters in 88 cases of lung cancer

*Variable*	n	*Low KCTD12*	*High KCTD12*	P *value*
*Age (years)*				
⩽55	22	13	9	
⩾55	66	31	35	0.324
				
*Gender*				
Female	40	21	19	
Male	48	23	25	0.668

*Tumor size (cm)*				
≤5	60	38	22	
⩾5	28	6	22	0.00025
				
*T-stage*				
1/2	68	38	30	
3/4	20	6	14	0.041
				
*N-stage*				
N0	36	20	16	
N1	35	19	16	1
				
*M-stage*				
M0	86	44	42	
M1	1	0	1	0.214
				
*Pathologic stage*				
Stages I and II	62	34	28	
Stages III and IV	26	10	16	0.160
