# Supplementary material for: KCTD12 promotes tumorigenesis by facilitating CDC25B/CDK1/Aurora A-dependent G2/M transition
Source: Oncogene. 2017 Sep 4;36(44):6177–89. doi: 10.1038/onc.2017.287 (PMC5671937; doi:10.1038/onc.2017.287)
Supplement: Supplementary Information [file onc2017287x1.pdf]

Supplementary Table S1. Differentially expressed proteins between M phase and S phase in HeLa cells.

ID	Protein_Name	Fold change (M/S)
P14635	G2/mitotic-specific cyclin-B1	9.11942
Q9NR50	Translation initiation factor eIF-2B subunit gamma	6.697576
Q63ZY3-2	KN motif and ankyrin repeat domain-containing protein 2	3.58971
P08670	Vimentin	2.983188
Q14739	Lamin-B receptor	2.460633
P07339	Cathepsin D; Cathepsin D light chain; Cathepsin D heavy chain	2.404639
O14974	Protein phosphatase 1 regulatory subunit 12A	2.398944
E9PCT1	Serine/arginine repetitive matrix protein 1	2.330071
**Q96CX2**	**BTB/POZ domain-containing protein KCTD12**	**2.110375**
G8JLA8	Transforming growth factor-beta-induced protein ig-h3	1.993274
P31942	Heterogeneous nuclear ribonucleoprotein H3	1.869595
P52732	Kinesin-like protein KIF11	1.76965
O00567	Nucleolar protein 56	1.727572
P48960	CD97 antigen; CD97 antigen subunit alpha; CD97 antigen subunit beta	1.713247
O43854	EGF-like repeat and discoidin I-like domain-containing protein 3	1.704087
Q92522	Histone H1x	1.691654
Q9NTZ6	RNA-binding protein 12	1.679673
P51991	Heterogeneous nuclear ribonucleoprotein A3	1.675949
P18858	DNA ligase 1; DNA ligase	1.655859
P22626	Heterogeneous nuclear ribonucleoproteins A2/B1	1.627623
P26006-1	Integrin alpha-3; Integrin alpha-3 heavy chain; Integrin alpha-3 light chain	1.619898
P14866	Heterogeneous nuclear ribonucleoprotein L	1.618746
Q49A26	Putative oxidoreductase GLYR1	1.617172
P20700	Lamin-B1	1.576901
P10909-2	Clusterin; Clusterin beta chain; Clusterin alpha chain; Clusterin	1.575963
Q8IY81	Putative rRNA methyltransferase 3	1.571082
P22087	rRNA 2-O-methyltransferase fibrillarin; rRNA/tRNA 2-O-methyltransferase fibrillarin-like protein 1	1.568467
Q96EA4	Protein Spindly	1.554616
Q13435	Splicing factor 3B subunit 2	1.547653
Q9UBW8	COP9 signalosome complex subunit 7a	1.543287
P02545	Prelamin-A/C; Lamin-A/C	1.530689
Q9P2N5	RNA-binding protein 27	0.649548

P18031	Tyrosine-protein phosphatase non-receptor type 1	0.646643
Q3KQV9	UDP-N-acetylhexosamine pyrophosphorylase-like protein 1	0.644095
Q9H307	Pinin	0.640999
E7EVA0	Microtubule-associated protein; Microtubule-associated protein 4	0.631046
Q8NF37	Lysophosphatidylcholine acyltransferase 1	0.623867
O60437	Periplakin	0.616209
H9KV28	Protein diaphanous homolog 1	0.605833
P61978-2	Heterogeneous nuclear ribonucleoprotein K	0.590422
Q9Y6G9	Cytoplasmic dynein 1 light intermediate chain 1	0.572449
P62140	Serine/threonine-protein phosphatase PP1-beta catalytic subunit; Serine/threonine-protein phosphatase	0.572188
Q96TA1	Niban-like protein 1	0.546986
Q08945	FACT complex subunit SSRP1	0.542808
Q8NB90	Spermatogenesis-associated protein 5	0.540596
O43491	Band 4.1-like protein 2	0.533298
P46821	Microtubule-associated protein 1B; MAP1 light chain LC1	0.523737
Q96T88-2	E3 ubiquitin-protein ligase UHRF1	0.510707
Q13618	Cullin-3	0.492311
P33991	DNA replication licensing factor MCM4	0.464841
Q09666	Neuroblast differentiation-associated protein AHNAK	0.455315
P29966	Myristoylated alanine-rich C-kinase substrate	0.450045
P57088	Transmembrane protein 33	0.338399
Q9C0C2	182 kDa tankyrase-1-binding protein	0.417544

Supplementary Table S2. Identification of KCTD12-interacting proteins by immunoprecipitation-mass spectrometry.

Entrez Gene name	Symbol
Protein SET	SET
ATP-dependent Clp protease ATP-binding subunit clpX-like, mitochondrial	CLPX
Cyclin-dependent kinase 1	CDK1
Poly [ADP-ribose] polymerase 1	PARP1
Galectin-3-binding protein	LGALS3BP
Twinfilin-1	TWF1
Splicing factor 3A subunit 3	SF3A3
RNA-binding protein with serine-rich domain 1	RNPS1
Endoplasmic reticulum-Golgi intermediate compartment protein 1	ERGIC1
Mitochondrial import receptor subunit TOM22 homolog	TOMM22
ATPase family AAA domain-containing protein 3A	ATAD3A
Proliferation-associated protein 2G4	PA2G4
RAC-gamma serine/threonine-protein kinase	AKT3
Pyruvate dehydrogenase E1 component subunit beta, mitochondrial	PDHB
Glutathione S-transferase Mu 3	GSTM3
Transmembrane emp24 domain-containing protein 10	TMED10
Ras-related protein Rab-7a	RAB7A
Arginine--tRNA ligase, cytoplasmic	RARS
Ras-related protein Rab-14	RAB14
Eukaryotic translation initiation factor 3 subunit I	EIF3I

Supplementary Table S3. siRNA sequences.

siRNA	Target sequence
si-KCTD12#1	5’-GGACAGCACACGTGGATTATT-3’
si-KCTD12#2	5’-GCAAAGGCCGCTTCTTTCTTT-3’
si-Aurora A#1	5’-TTCTTAGACTGTATGGTTATT-3’
si-Aurora A#2	5’-AATAGGAACACGTGCTCTATT-3’
si-CDC25B#1	5’-GGAAAAGGACCTCGTCATGTT-3’
si-CDC25B#2	5’-AGACTGCAGATACCCCTATTT-3’
si-Control	5’-TTCTCCGAACGTGTCACGTTT-3’

Supplementary Table S4. Primers for site-directed mutagenesis.

Plasmid	Primer
pCMV-N-flag-KCTD12 S185G	Forward: 5’-CTGGAGCTGGCTAGCCGCGGTCCGTCCGG-3’
	Reverse: 5’-CGCGGCTAGCCAGCTCCAGCGTGGGCGAC-3’
pCMV-N-flag-KCTD12 S187A	Forward: 5’-CTGGCTAGCCGCAGTCCGGCCGGGGGCGC-3’
	Reverse: 5’-CCGGACTGCGGCTAGCCAGCTCCAGCGTG-3’
pCMV-N-flag-KCTD12 S200A	Forward: 5’-CTGCTCACGCCGTCCCAGGCGCTGGACGG-3’
	Reverse: 5’-CCTGGGACGGCGTGAGCAGCGGGCCCGCC-3’
pCMV-N-flag-KCTD12 S243A	Forward: 5’-TGCGGAAAGACGGCGCTGGCCAAGGAG-3’
	Reverse: 5’-CTCCTTGGCCAGCGCCGTCTTTCCGCA-3’

Supplementary Figure legends

Supplementary Figure S1. KCTD12 promotes lung cancer cell proliferation. (**a–b)** A549 cells were transfected with a KCTD12-expressing plasmid (1 μg, 24 h) or an empty vector. KCTD12 expression was assessed by western blotting (**a**), and the colony formation ability of the above cells was compared by colony formation assay (**b**). (**c-d)** KCTD12 knockdown inhibits cell proliferation in A549 cells. Bars, SD; **, $P < 0.01$; ***, $P < 0.001$ compared with control cells.

Supplementary Figure S2. KCTD12 is phosphorylated by Aurora A in M phase. (**a)** Western blotting analysis of KCTD12 expression in HeLa cells synchronized or unsynchronized to M phase. Note that the upward-shifted band of KCTD12 observed in M phase cells disappeared when the cell lysate was treated with CIP (10 U/ml cell lysate). (**b)** The upward shifted band of KCTD12 was also abolished in cells treated with VX-680 (0.5 μM, 24 h). Red arrow, upward-shifted KCTD12 band.

Supplementary Figure S3. Serine 200 is not required for the cancer-promoting effects of KCTD12 in HeLa cells. **(a-b)** HeLa cells were transfected with a plasmid expressing wild-type KCTD12, a KCTD12 S200A mutant, or an empty control (**a**), and colony formation assay was performed to compare the ability of these cells to form colonies (**b**). Bars, SD; *, $P < 0.05$ compared with the cells transfected with the KCTD12-expressing plasmid; n.s, no significance.

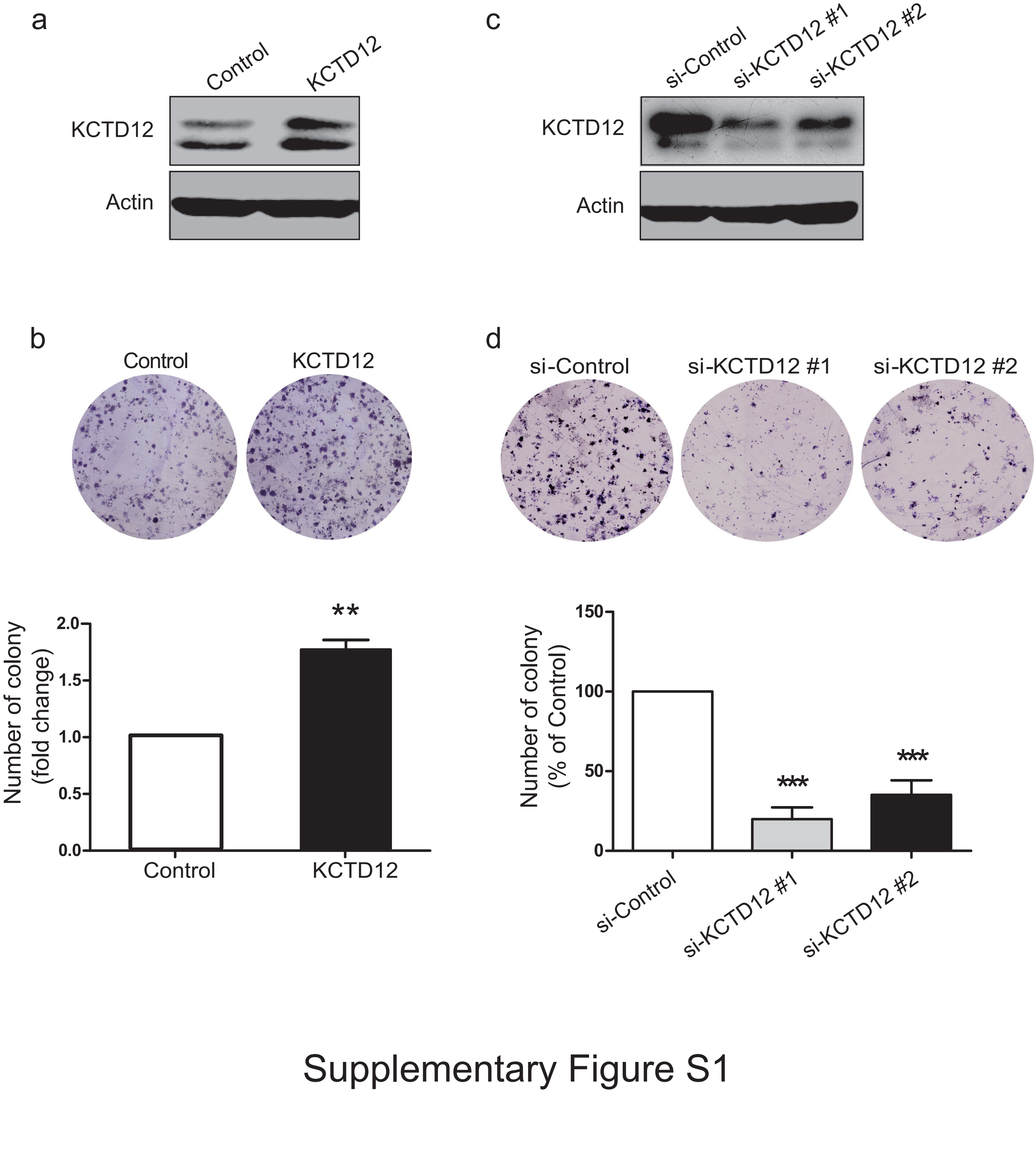

Supplementary Figure S1

a

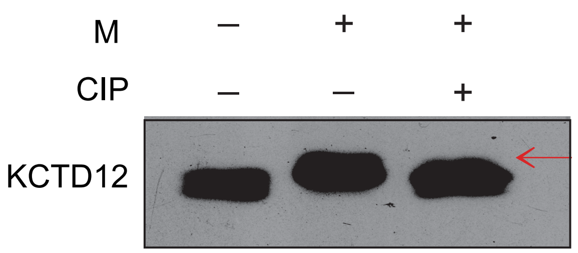

b

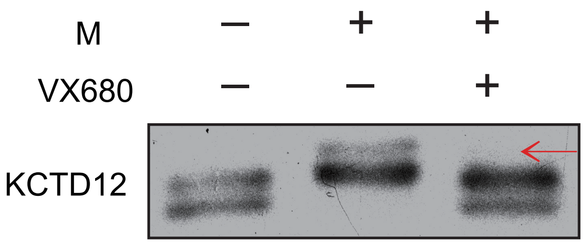

Supplementary Figure S2

a

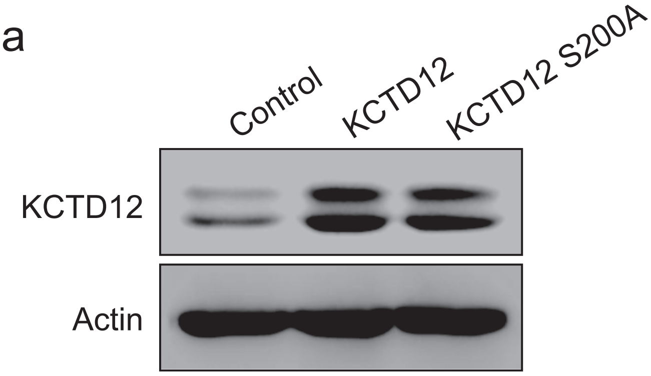

b

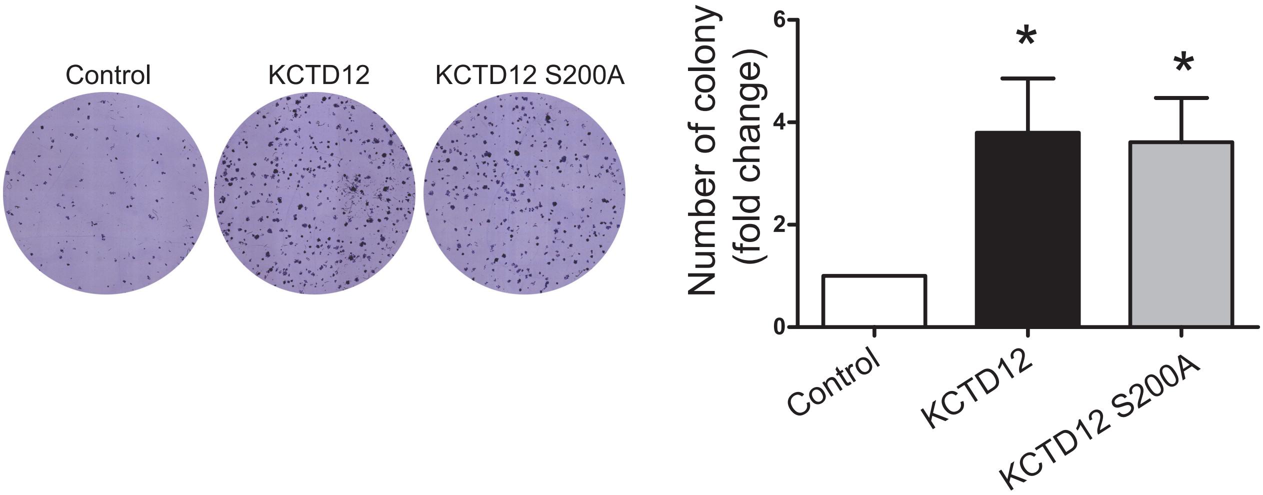

Supplementary Figure S3
